# Derby County Asylum

**Published:** 1853-07-01

**Authors:** 


### DERBY COUNTY ASYLUM.

It appears from the first Report of this Asylum that, so far back as the year 1844, an estate consisting of seventy acres of land at Mickleover, three miles from Derby, was bought, and designs were adopted and matured, and every preparation made to accept tenders for the works. But as there were reasons to expect the early appointment of a new Commission in Lunacy, from which the committee might reasonably hope to obtain valuable advice and facilities for effecting more economical financial arrangements, especially with respect to the repayment of monies borrowed on the security of the County Rates, they deemed it prudent to suspend their proceedings until the Commission should be appointed, and they could have the benefit of acting with and under it. However, an impression then prevailed that a Lunatic Asylum might be erected for much the same cost as a Union Poor-House, the essential differences between the two institutions, and the capacity, habits, and necessities of the inmates being considerably overlooked. The plans submitted by the committee were the result of much and anxious consideration, of personal enquiry made by three members of the body who visited, for this special purpose, many of the best existing Asylums, and of the advice and assistance of one of the ablest and most distinguished managers of such Institutions. The plans were first laid before the new Commissioners who made the general objection, that the estimate was too high and the accommodation too great, but without stating or giving us any means of judging what, in their opinion, would be a proper estimate, or a proper number to be accommodated. This led to a long discussion and correspondence, which ultimately terminated at the end of the year 1847, in the adoption, by the Commissioners, of a design essentially identical in principle with that first submitted to them, but with a reduction in the number of patients to be accommodated from 360 to 300.

The Asylum, says the Report, is situated about three quarters of a mile S.W. of the village of Mickleover, on an estate consisting of seventy-nine acres of land, purchased by the county for the purpose of the institution, and stands, with a southern aspect, on rising ground overlooking the rich valley of the Trent, and commanding a panoramic view of the wide spread country beyond, bounded in the extreme distance to the south, by the Charnwood hills, having Needwood to the west, and the flats of Nottinghamshire to the east—perhaps one of the most beautiful and varied views in the country. The property is approached from the Uttoxeter road by a pleasant lane, which forms its northern boundary, and in which, at the N.E. angle of the estate, are the entrance gates with a lodge attached; a carriage drive, with turfed slopes, leads from these gates past the eastern front, and along the south terrace which stretches across the property from east to west, to the CHIEF ENTRANCE in the centre of the southern or principal front, the drive being continued forward to the farm building at the N.W. boundary. The farm is also approached by a back road from the entrance lodge, which, striking the plantation (about four acres in extent to the north,) also affords access to the offices and back premises of the establishment. A kitchen garden (containing about five acres) surrounds the building on the north, east, and west sides, and occupies the remaining space enclosed by the roads before-mentioned. The site of the building and exercise gardens covers a space of 5½ acres.

We regret that we are, from want of space, unable to extract more fully from the minute account given in the document before us, of the general arrangements of this institution. Dr. Hitchman's report is an excellently drawn up account of the medical progress of the Asylum since it was opened for the reception of patients. He says :—

"On the 21st of August, 1851, the first patient was admitted into this institution. The Asylum was in a very unfinished condition, but the various parishes in the county having been led to expect its completion at that time, and the Union of Belper being under the necessity of sending a patient to London, if he could not be admitted into this Asylum, permission was given for his reception, and he was admitted accordingly. Your physician was informed that the patient had not been long ill, but he proved to be both epileptic and paralysed, and required all the resources of a well-appointed hospital. One ward in each wing of the asylum being completed and furnished, other individuals were speedily admitted. It may be gratifying to the committee to learn, that the first group of patients who were brought from various workhouses and asylums, were delighted with their new abode, and especially with the airing grounds; in these they spent much time, pointing out to each other distant objects in the landscape, and gathering enjoyment from the beauty, the quietude, and extent of the surrounding scenery. The general expression of their countenances, and their language indicated this, and none with greater force than a paralytic, who when brought to the asylum was carried by an attendant from a cart to the wards, being unable to walk from emaciation and debility, and from deep excoriations, and sloughing ulcers on the hips, sacrum, shoulders and knuckles, produced by the combined effects of paralysis and mechanical restraints. This patient was brought in a large and easy chair to view the scene; he was apparently listless and stupid, and yet when he had been a short time in the warm open air, surrounded by cheerful companions, and possessing a full view of the green meadows, the distant hills, and all those varied hues of light and shade which filled up the landscape before him, his mind awoke from its lethargy, and with animated pathos, the poor fellow said, 'It is a nice place, I hope I may live to enjoy it.' From this time he ceased to be moody and became hopeful, his physical health improved, his wounds healed, he became a useful member of the household, and continued so for several months until a severe attack of apoplexy incapacitated him for exertion. Other patients in their letters to their friends make frequent, and touching allusions to the beauty of the spot on which the asylum is placed, and some of the better educated attempt to embody in verse the pleasure it brings to them. These facts are narrated to illustrate a principle, and to show the importance of surrounding the insane with cheering and soothing objects."—pp. 18, 19.

When referring to the form of the asylum, and after paying a just eulogium to the genius of the architect, Mr. Duesbury, Dr. Hitchman says,—"The central position of the main offices, the entire privacy of each ward, which has been secured by short and well arranged corridors of communication, the facility of access and supervision, the arrangement of the sleeping rooms, the number and character of the baths and padded rooms, the mode of warming the galleries, the length and breadth of the wards, their cheerful character, the perfect manner in which, by means of a large window at the end of each gallery and the falling back of a ward, the pleasing features of the surrounding country are secured equally to the entire asylum, the height and structure of the building, and the arrangements of its walls and airing courts are in greater harmony with the teachings of that distinguished philanthropist, than are those of any asylum which preceded it, or indeed of any other, with which your physician is acquainted."

In relation to the statistics of the asylum, Dr. Hitchman observes,—"Since the opening of the asylum in August, 1851, two hundred and twelve patients have been admitted; of these, one hundred and sixteen were men, and ninety-six were women. The following Table will show the relative proportion of single and married:—

| | Men. | Women. | Total. |
|---|---|---|---|
| Single | 51 | 35 | 86 |
| Married | 51 | 48 | 99 |
| Widowed | 12 | 12 | 24 |
| Unascertained | 2 | 1 | 3 |
| | 116 | 96 | 212 |

"In reading the above particulars, it must be borne in mind, that no safe conclusions can be drawn from statistics embracing so small a number of facts. Thus, the Table coincides with the deductions of large numbers, as regards the relative liability of the two sexes to insanity, but differs from those which have been obtained as to the comparative liability of the married and single to this fearful malady.

"As might have been expected in a pauper class, the majority of the patients are uneducated. The following Table conveys an imperfect idea of the extent of this condition, because as in similar tables published at Hanwell and elsewhere, every person is placed under the category of being able to write, who can form writing characters, however slowly and imperfectly he may write out a sentence; and among those who can read are placed, not only those who do so with fluency, but those also, who can by prolonged effort read a few sentences:—

| | Men. | Women. | Total. |
|---|---|---|---|
| Can read and write | 61 | 32 | 93 |
| Can read only | 25 | 37 | 62 |
| Unable to read | 30 | 27 | 57 |
| | 116 | 96 | 212 |

"The following Table will furnish a summary of the admissions, discharges, and deaths, which have taken place since the opening of the asylum:—

| | Men. | Women. | Total. |
|---|---|---|---|
| Patients admitted | 116 | 96 | 212 |
| Discharged recovered | 17 | 17 | 34 |
| ——— Improved. | 1 | 2 | 3 |
| ——— Unimproved | 4 | 1 | 5 |
| Escaped | 1 | 0 | 1 |
| Died | 9 | 7 | 16 |
| Out on trial | 0 | 1 | 1 |
| Total, discharged, escaped, and died | 32 | 28 | 60 |
| Remaining in the asylum, 1st Jan., 1853 | 184 | 68 | 152 |

"Of the above two hundred and twelve patients, one hundred and eighty-one were in a chronic state at the period of their admission, and among them twenty-three were epileptic, twenty more or less paralysed, and nine were congenital idiots. From this collective number of 181 six only have been discharged cured. Let us look at the other side of the picture. Thirty-one cases uncomplicated with old age, epilepsy, or paralysis, have been admitted at the onset of their mental derangement. Of these, twenty-eight have been discharged cured, and two of the others have been a short time under treatment, with every prospect of ultimate success. These facts speak for themselves. They proclaim with great force the importance of sending patients for appropriate treatment in the earliest stage of their malady.

In reference to the general character of the patients admitted Dr. Hitchman says,—"The majority of the patients admitted have not only been in a chronic state of insanity, but also in a feeble state of bodily health. In some of the cases, the mental disease appeared to have been caused by the want of a proper supply of healthful nutriment to the brain. Two women who were admitted, weighed only seventy-four pounds, and sixty-seven pounds respectively. The lighter of the two died, the other recovered—the means of cure consisting chiefly in the good dietary, the warmth, the cleanliness, and general cheerfulness of the establishment. It may be remarked here, that one of the most striking incidents in the progress of the cases, which have become well, has been the gradual increase in the weight of the patients as the mental disease subsided. During the paroxysm of mania, no matter how voracious the appetite, or how abundantly supplied, emaciation proceeded; but as convalescence returned, the individuals gradually increased in size and weight; and this process has, in some instances, gone on, even after the patient has left the asylum, so that as after an attack of fever, or other kindred maladies, the individual has become stouter than he had ever been before."

When speaking of the treatment of the epileptic patients, Dr. Hitchman remarks, "the tincture of sumbul, and the extract of the cotyledon umbilicus, have been employed during the past year, as remedies in this malady, but with no better success than usually attends a novel medicine in this disease. With the use of each drug, the fits appeared, for a short time, to be less frequent than before its administration; but this effect was equally marked when *any* medicine was given to them with an air of confidence. As a class, they are fond of physic and hopeful of cure."

The medical officer pays a just tribute of praise to the humanity and efficiency of the nurses employed in the asylum:—"Two female patients, in a state of frantic mania, and in an advanced state of pregnancy, have been admitted, and safely delivered of living children. These cases were full of embarrassment and danger, and required the greatest courage, forbearance, and kindness on the part of the nurses. From a combination of circumstances in one case, such as profuse hemorrhage after the birth of the child, accompanied by intense and prolonged maniacal phrenzy, the services of three nurses, and sometimes more, were required for several days and nights in succession. This case occurred during the time that Dr. Hutchinson Ramsay had the temporary charge of the asylum; and your physician is happy to testify, not only to the skill which was brought to bear upon this especial case, but also to the very efficient manner in which the other duties of the asylum were conducted during that gentleman's superintendence. Neither would he be doing justice to his own feelings, or to those of Dr. Ramsay, were he not to record the devotedness, the energy and industry which were displayed by the nurses of the establishment during these trying emergencies. Each nurse endeavoured to excel the other, in their efforts to control the patient, and to render such other aid by night and day, as the case required. Chloroform was, with great advantage, administered to the above patient, under the advice of Dr. Forbes Winslow, who happened to visit the asylum at that particular period."

The following case will illustrate the effects of kind and skilful treatment:—"T. G., removed from the custody of his relatives by the order of the magistrates. Has been insane thirty-eight years, under the management of his relatives, who have generally had him confined in an out-building. He is stated to have been unclothed for many years. When brought into the asylum he was naked, except that around his pelvis were the remains of an article of dress; his hands were tightly bound to each other by ligatures passing around the wrists. When in the cart he was covered with a blanket, but this fell from him during his struggles on being removed. He roared hideously as he was being conveyed to the wards. He is a person of lofty stature and great size. His head and neck are very large; one side of his forehead is greatly disfigured by scars, and he has lost an eye. His ears have been deprived of their normal shape, and their lobes much thickened by the deposition of fibrine or other matter. His lips are large and pouting. His beard has been long unshaven, but has been recently cut with a pair of scissors. The bones and muscles of his arms are of great size—his lower extremities are red, swoln, and 'pit,' under pressure; one of his toes is deprived of its nail, and the whole foot appears to have suffered from the effects of cold. He walks with a stooping gait, and appears unable to retain the erect posture without support. He resists powerfully all attempts to clothe him; and appears to be entirely ignorant of the use of a bedstead. He whines after the manner of a dog that has lost its home. He dreads all who approach him; on being taken from his room in the evening, he hurried back to it with all the haste he could, and on all occasions he shrinks from observation. He is lost to every sense of decency; nakedness is congenial to him, but he will sometimes coil himself in a blanket for the sake of its warmth. He is guided by the lowest instincts only, and his whole appearance and manner, his fears, his whines, his peculiar skulking from observation, his bent gait, his straight hair, large lips, and gigantic fore-arm painfully remind one of the more sluggish of the Anthropoid apes, and tell but too plainly to what sad depths the human being can sink under the combined influence of neglect and disease."—Case Book, page 43.

Fifteen months have elapsed since the admission of the above patient—he now walks about the galleries properly clothed, smiles when he is approached, puts out his hand in a friendly manner towards those he recognises, sits regularly at meals, is shaved at appointed times, carries himself nearly erect, and looks as if he belongs to the children of men.

The "non-restraint" system of treatment, Dr. Hitchman says, "has been severely tested during the past year. Patients have been brought to the asylum in every kind of restraint, and with most fearful characters; but no amount of fury or of strength, and no description of character, however terrible, have caused us to hesitate for a moment in freeing the patient from his cords and fetters, and in no single instance has there been reason to regret the proceeding. The case of T. G., confined for many years in an outhouse—of A. G., and J. T., are illustrations; but the following particulars of a case very recently admitted, will serve to illustrate the principle: '—— Workhouse, December 18, 1852.—He has been with us about twenty-four hours, and a *terrible* night we have had with him. He has been shouting '*Murder*' at the top of a stentorian voice, enough to alarm the neighbourhood for some distance. One of his attendants he knocked down, and we found it to be absolutely necessary to restrain him. Gloom and melancholy appear to be a phase of his insanity, with alternate violence, and certainly at these times he appears to have the strength of an elephant.' Such was the note of introduction which accompanied the patient. One of three men who brought him stated that his finger had been severely crushed by the patient, who pinioned him between the door and its post, 'and kept me there for more than an hour.' If ever 'restraint' was needful it was with this man. He is six feet high, very muscular, and with a wrist which few persons can span. He was brought to the asylum firmly pinioned by ropes and hand-bolts, and his arms were severely bruised from this cause. In a few minutes all the manacles were removed; he has had the perfect use of every limb since he has been in the asylum, and has been fully controlled by moral means alone."

We extremely regret that our limited space compels us to omit several other portions of this valuable report, which we had marked for insertion. We hope all who feel interested in the progress of an enlightened treatment of the insane, will procure this report, and "mark, read, learn, and inwardly digest" its contents. We have placed as a frontispiece to this number of our journal, a lithographic sketch of this institution.

